# Range of Motion and Intensity Achieved During a Single Session of Targeted Robot-Assisted Exercise in Individuals with Parkinson’s Disease: A Pilot Study

**DOI:** 10.3390/s26041091

**Published:** 2026-02-07

**Authors:** Meredith D. Wells, Matthew Lamsey, Arielle Wallenstein, Jerry Feldman, Charles C. Kemp, Madeleine E. Hackney

**Affiliations:** 1 Division of Geriatrics and Gerontology, Department of Medicine, Emory University School of Medicine, Atlanta, GA 30329, USAawalle7@emory.edu (A.W.); 2 Institute for Robotics and Intelligent Machines, Georgia Institute of Technology, Atlanta, GA 30322, USA; lamsey@gatech.edu; 3 The Parkinson’s Foundation, New York, NY 10018, USA; 4 Hello Robot, Inc., Martinez, CA 94553, USA; 5 Atlanta VA Health Care System, Rehabilitation R & D Center, Decatur, GA 30033, USA; 6 Department of Veterans Affairs Birmingham/Atlanta Geriatric Research Education and Clinical Center, Brookhaven, GA 30319, USA; 7 Department of Biomedical Engineering, Georgia Institute of Technology, Atlanta, GA 30332, USA

**Keywords:** rehabilitation, biomechanics, sensors, inertial measurement units, mobility

## Abstract

The goal of this study was to determine if a robot-assisted exercise system could lead individuals with Parkinson’s disease (PD) through different joint ranges of motion in a fun and effective manner. Eleven individuals with PD participated. A novel robotic system placed a target at different places in space for participants to tap with their hand, foot or knee. The range of motion (ROM) was collected by inertial measurement units (APDM), and was extracted using a custom code (Matlab). ROM was dependent upon the exercise and joint of interest. Participants illustrated acceptable levels of fatigue during each session, based on an average ending heart rate of 107.0 ± 11.9 bpm (~70% of maxHR) and an ending RPE of 6.5 ± 1.8 on a 10-point scale, indicating that the sessions were appropriately challenging. Standing forward reach, used to assess static balance and flexibility, improved by an average of 1.7 inches (*p* < 0.01), demonstrating immediate improvements from exercising with the robot. The results demonstrate the potential benefits of exercising with a robotic exercise system. The number of sessions spent with a PT can be limited by availability, so this system could be a fun way to encourage individuals with PD to complete their PT exercises at home.

## 1. Introduction

Globally, Parkinson’s disease (PD) is the fastest growing neurodegenerative disease [1]. Affecting one percent of individuals over the age of 60 and four percent of individuals over the age of 80, PD is the second most prevalent neurodegenerative disease [2]. Approximately 10 million people worldwide are living with PD [1].

PD is a complex, progressive, neurodegenerative disease [3] that causes a variety of motor deficits resulting from a loss of dopaminergic cells in the basal ganglia [4]. In the early stages of PD, patients display four cardinal motor symptoms: bradykinesia, gait alterations, rigidity, and tremor. With bradykinesia, individuals experience slowness of spontaneous movement and reduced speed and amplitude of voluntary movements. Gait alterations may appear as a decreased arm swing, dragging of one leg, and a hunched posture. Rigidity is the term used to describe increased muscular tension. Tremor presents as a shaking or involuntary movement and is typically observed in the chin, jaw, limbs, and lips. These motor symptoms tend to persist into the later stage and are accompanied by freezing of gait and postural alterations [5]. There is a >75% prevalence of dementia in individuals who have had PD for ten years or longer [6]. Such cognitive decline may be associated with a poor ability to undertake dual tasks, i.e., the ability to pay attention to and complete two tasks at once, such as walking and counting [5,7].

Treatment for PD tends to vary based on the symptoms a patient is experiencing, but it often involves some combination of pharmacological and non-pharmacological components. The gold standard for treating PD pharmacologically is with dopamine precursors, such as levodopa [8]. Individuals taking levodopa for prolonged periods almost universally experience a phenomenon known as the “on–off” cycle. This side effect is characterized by fluctuations in an individual’s psychomotor condition from periods of immobility and depression to periods of higher mobility and energy every couple of hours [9]. Non-pharmacological interventions used to manage PD symptoms include invasive approaches such as deep brain stimulation and non-invasive therapies such as physical therapy [10].

Physical therapy (PT) has been demonstrated to slow the functional decline of balance, gait speed, and physical functioning in individuals with PD by improving their range of motion (ROM) and increasing their strength [11]. A prior study demonstrated that people with PD who engage in physical exercise have a lower rate of mortality [12]. This evidence underscores the transformative potential of PT in managing PD, offering significant improvements in mobility, quality of life (QOL), and longevity.

Common focuses of PT include cardiovascular health, and resistance, balance and flexibility training [13]. Cardiovascular exercise yields both immediate and long-term benefits for PD symptoms. One session of structured treadmill training has been shown to improve gait parameters such as speed and stride length [14]. Another study showed that a single cycling session improved bradykinesia and tremors in people with PD [15]. When cardiovascular exercise is performed consistently over the course of a few weeks to a few months, participants report improved mobility, reduced gait impairments, reduced fall risk and fear of falling, improved QOL, and reduced postural instability [16,17]. Despite the significant benefits associated with PT, there are some limitations, such as therapist shortages [18]. As a result, patients frequently fail to achieve the full benefits of PT due to the limited time physical therapists can dedicate to each individual patient. Therefore, it is critical to investigate the means of providing PT to individuals with PD without the need for a therapist to be consistently present.

The gap between the availability of supervised PT sessions and the amount of therapy required to maximize the benefits has led to the introduction of robotic systems into PT for certain populations. A recent systematic review and meta-analysis on robot-assisted training and people with Parkinson’s disease reported some success with rehabilitation outcomes related to lower limb function, fatigue, and balance confidence, while there were no significant improvements in upper limb mobility [19]. Yet, there are a few drawbacks to the prior attempts to utilize robot-assisted devices in PT, including the lack of portability of the devices, discomfort, and boredom, all of which can lower the rate of adherence to the exercise protocol [20]. In addition, exoskeleton robots, though a powerful rehabilitative tool for many individuals, are cumbersome to don and remove, and can be expensive and dangerous to operate. While robotic integration in PT shows promise for enhancing patient engagement and outcomes, its effectiveness relies on designs that remain accessible and intuitive for older adults with mobility and dexterity challenges. Thus, it is critical that the technologies are user friendly.

The goal of this study was to determine if a robot-assisted exercise system could effectively lead individuals with PD through ROM at different joints, provide a moderate workout, and improve balance and mobility immediately after a single session. It was hypothesized that the custom-designed exercise system would encourage participants to move through different exercises, would be moderately physiologically challenging, and would result in immediately improved balance and mobility, as assessed by a standing forward reach task.

## 2. Materials and Methods

### 2.1. Participants

A total of 11 individuals with idiopathic PD (7 female; age: 68.2 ± 6.2 yrs; time since diagnosis: 7.1 ± 5.1 yrs; Hoehn &Yahr (H&Y) stage: I–II.5) participated in this study. All participants could walk 10 feet independently, either with or without an assistive device. The exclusion criteria included a diagnosis of dementia, vascular cognitive impairment, memory deficits, or other neurological disorders. This study protocol was approved by the Institutional Review Boards at Emory University and the Georgia Institute of Technology (STUDY00004909), and all participants signed a written informed consent document prior to participating.

### 2.2. Robotic Physical Therapy System

**Figure 1:**
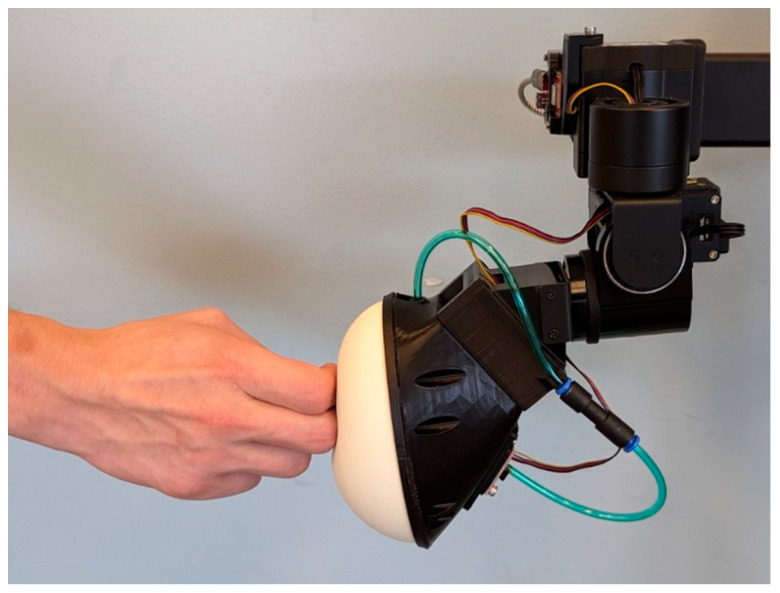
The soft-bubble end effector registers contact by detecting changes in the bubble’s internal pressure when pressed on by participant’s hands, knees, and feet. The bubble provides congruent haptic and auditory feedback by playing a sound effect upon contact detection.

**Figure 2:**
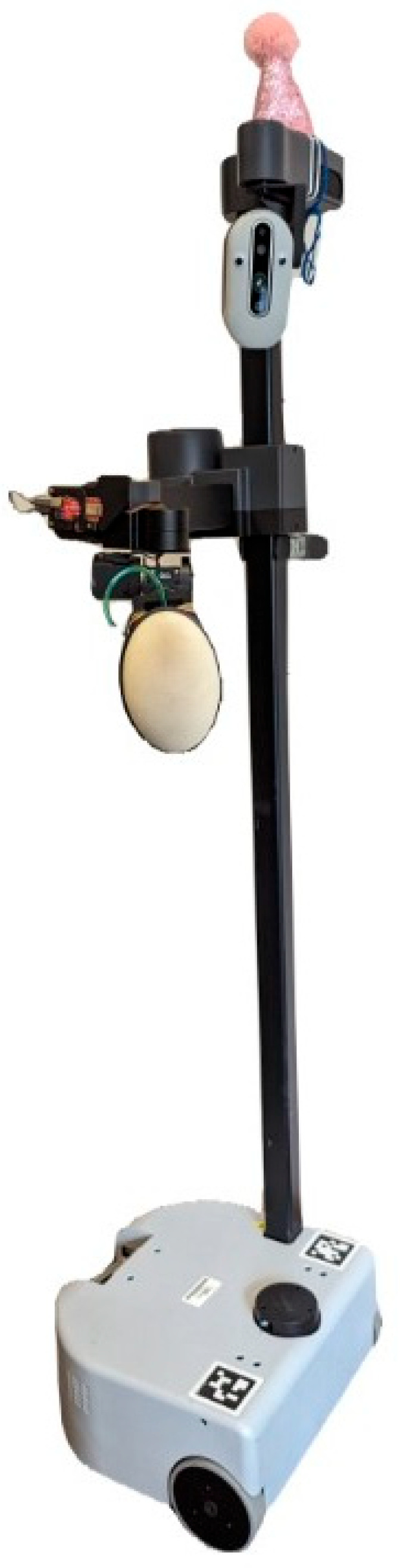
The Stretch-with-Stretch (SWS) system consists of a mobile manipulator with a soft-bubble end effector which serves as an external cue for participants to repetitively reach towards and press on with different parts of their body, such as their hand or foot. The location of the bubble, relative to the participant, is personalized for each exercise.

This study utilized a novel interactive robotic system called Stretch with Stretch (SWS) (Hello Robot, Inc., Martinez, CA, USA) [21], which uses a Stretch RE1 mobile manipulator [22] that was modified to have a pressurized soft bubble end effector [23]. The soft bubble (Figure 1) served as a haptic target for participants to reach towards and press on with their hands, feet, or knees while completing each exercise repetition. Contact with the bubble by the participant is detected by changes in the internal pressure of the bubble, allowing the robot to count each repetition. The robot’s workspace extends from the floor to approximately 1.1 m above the floor, its arm extends 0.5 m, and its mobile base can drive around participants, thereby enabling diverse target placement for a variety of stretching exercises (Figure 2).

The location of the bubble target for each exercise was personalized for each participant, within parameters that were previously discussed in [21]. SWS guided each participant through a calibration process that allowed for the determination of the target’s starting point for each exercise [21]. During the exercise session, SWS also modified the exercise difficulty level, or how far away the target is from the participant, after every exercise set, based on the performance during the first set of each exercise.

SWS was programmed to provide verbal instructions for each exercise and was connected to an external monitor for visual demonstrations via pre-recorded videos. Following each exercise set, SWS provided the participant with a score based on how many times the participant contacted the bubble, and a congratulatory phrase both during and following each exercise set. SWS also leveraged speech recognition software to track the verbal responses captured with a lapel microphone during the dual-task exercises.

### 2.3. Protocol

Upon arrival at the lab, participants completed questionnaires regarding their quality of life and overall health, PD symptoms, and balance and physical activity levels. Participants then completed 6 motor–cognitive tasks designed to assess their movement and cognitive capacities. These tasks included the 30 s chair stand [24], the body position spatial task [25], the four-square step test [26], a timed up-and-go with and without the cognitive aspect [27], and a 6-min walk test. Additionally, participants completed a standing forward reach test before and immediately following the robotic exercise session to determine if there were any immediate effects on their balance and flexibility. The standing forward reach task was completed by asking the participants to stand next to a wall and to raise their left arm (the one closest to the wall) to shoulder height. They then aligned their fingertips with a piece of tape on the wall, which designated the starting point for each repetition. Participants were then instructed to bend at the waist and lean as far forward as possible without losing their balance, such that their left arm slid along the wall. Once participants could not reach any further, the ending location of their fingertips was marked for measurement purposes. The task was completed 3 times before and 3 times at the conclusion of the robot-assisted exercises. The best score was used each time.

Prior to beginning the robot-assisted exercise session, participants were outfitted with a chest strap heart rate monitor (Polar Electro, Kempele, Finland) to collect objective data on the exercise intensity level, and 15 Opals inertial sensors (Opals, APDM Wearable Technologies, Portland, OR, USA) were used to collect ROM information for each exercise throughout the session. Opals IMU sensors were chosen for this study due to the space and resource limitations, ease of instrumenting participants, and because of their clinical validation and use in previous related research [28,29]. The exercise session consisted of 6 exercises—3 seated followed by 3 standing. The seated exercises included a forward reach, and two exercises that required gastrocnemius activation: knee extension and calf raise, in that order. The standing exercises emphasized contrabody movement, which is often impaired in PD, and included a horizontal cross-body reach; windmills, where they were asked to reach diagonally downward to the opposite ankle; and high knees, in that order. The exercises were always executed in the same order. The calf raises and the horizontal cross-body reach both included a cognitive dual task component, where participants were asked to list a different U.S. state with each repetition and a different animal with each repetition, respectively. Cognitive answers were allowed to be repeated between sets, but individuals were asked to list unique answers within each set as best as possible.

For each exercise, SWS provided verbal instructions accompanied by a video and then conducted a calibration to determine an appropriate starting position for each participant and task. The participant was then given a 10 s trial to ensure they understood the provided instructions. This practice was followed by two 30 s sets of the exercise on the right side and then the left side for a total of four 30 s sets per exercise. Between the first and second set on each side, SWS would modify the difficulty of the starting position for the target, based on performance during the first set. This sequence was repeated for each of the 6 exercises. Rest was built in as SWS repositioned itself, provided feedback, and gave instructions, but additional rest was provided as requested.

For participants to utilize an exercise system consistently, it is important that they enjoy using it and they see a benefit from it. Therefore, following the entire exercise session, participants were asked if they would find a system like SWS useful in their daily exercise. Participants were also asked to report their rate of perceived exertion (RPE), or how hard they felt they were working, before, during, and after the exercise system.

### 2.4. Statistical Analysis

**Figure 3:**
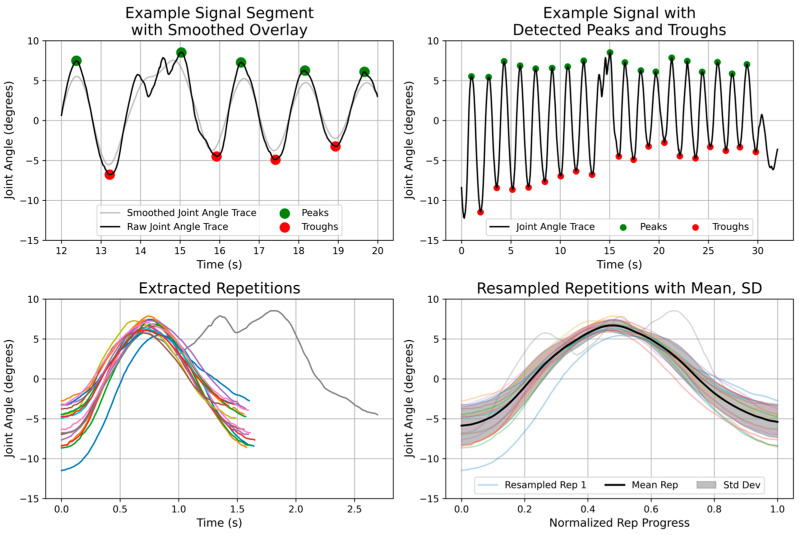
Signal processing and range of motion extraction on one set of one participant’s performance of the seated forward reach exercise. Raw joint angle time-series data are first smoothed, using a Savitzky–Golay filter, to identify plausible locations of local extrema (**top left**). Peaks and troughs corresponding to individual exercise repetitions are then detected using a minimum peak–peak separation criterion (**top right**). For each detected extremum, the true maximum or minimum is refined by selecting the corresponding extremum from the unsmoothed signal within a ±0.25 s window centered on the candidate location. Individual repetitions are segmented between successive extrema and temporally aligned (**bottom left**), then resampled onto a normalized time axis to enable the computation of the mean repetition profile and standard deviation envelope (**bottom right**), which are used to estimate joint-level ROM and inter-repetition variability. The colored lines in the bottom figures represent individual repetitions.

A MATLAB-based data processing pipeline (MATLAB R2023b, MathWorks, Natick, MA, USA) was used to estimate each joint’s ROM for each trial of each exercise. Joint angle wraparound errors in the Opals recordings were first corrected. Plausible locations for ROM extrema were then identified on a smoothed version of each time series, using a third-order Savitzky–Golay filter (window length 71–201 samples, depending on the exercise). Local minima and maxima were detected using MATLAB’s findpeaks() function with a minimum peak–peak separation of 0.4 s. For each candidate extremum on the smoothed signal, the corresponding true extremum was refined by searching the unsmoothed signal within a ±0.25 s window centered on the candidate location and selecting the maximum or minimum value within that window. Finally, extrema pairs producing ROM values more than two standard deviations from the mean ROM were excluded as outliers. An overview of this process is provided in Figure 3.

Descriptive statistics (mean and standard deviation) were computed for the average and peak ROM data for each exercise set on each side, and the motor–cognitive assessments. A paired *t*-test conducted in Excel was also utilized to compare pre- and post-exercise standing forward reach scores.

## 3. Results

### 3.1. Demographics and Functionality

All participants successfully completed the protocol without any adverse events. Participant characteristics are presented in Table 1 and motor and survey pre-assessment scores are presented in Table 2. Overall, participants were moderately functioning and reported a generally high quality of life. They did have some balance impairment and reported an overall low fear of falling. For simplicity in reporting, the average and peak ROM data and the numbers of repetitions completed for each task have been averaged between the two trials for the right side and the two trials for the left side. Individual trial data for the right side can be found in Table 3, and individual trial data for the left side can be found in Table 4.

### 3.2. Range of Motion

During seated calf raises, participants moved through an average of 34.9 ± 15.0 degrees of plantarflexion at the right ankle, with a max ROM of 38.5 ± 25.8 degrees and an average of 22 ± 9 repetitions completed. On the left, participants averaged 33.2 ± 12.5 degrees of plantarflexion, a max ROM of 41.5 ± 13.0 degrees, and 23 ± 10 repetitions completed.

For the seated forward reach, participants demonstrated an average of 22.1 ± 9.6 degrees of hip flexion, a max hip ROM of 30.1 ± 14.5 degrees, and an average of 20 ± 2 repetitions on the right side. On the left side, participants demonstrated an average of 19.4 ± 8.7 degrees of hip flexion, a max ROM of 23.8 ± 10.5 degrees, and 21 ± 4 repetitions.

When performing the seated knee extension exercise on the right side, participants illustrated an average of 54.7 ± 20.9 degrees of knee extension, a max ROM of 66.3 ± 21.8 degrees, and an average of 23 ± 3 repetitions. On the left side, participants illustrated an average of 50.3 ± 11.6 degrees of knee extension and a max ROM of 53.0 ± 29.6 degrees, and averaged 22 ± 3 repetitions.

During the standing cross-body reach exercise with the right side, participants moved through an average of 9.5 ± 3.7 degrees of hip flexion and 15.9 ± 5.8 degrees of trunk rotation, a max ROM of 13.3 ± 7.0 and 18.4 ± 6.6 degrees of hip flexion and trunk rotation, respectively, and completed nine repetitions. On the left side, participants moved through an average of 8.8 ± 2.9 degrees of hip flexion and 14.3 ± 5.5 degrees of trunk rotation, had a max ROM of 13.0 ± 9.2 and 16.5 ± 6.6 degrees of hip flexion and trunk rotation, respectively, and completed seven repetitions.

For the standing windmills exercise, participants demonstrated an average of 58.9 ± 18.1 degrees of hip flexion and 28.3 ± 11.6 degrees of trunk rotation, a max ROM of 65.9 ± 17.9 and 33.7 ± 12.8 degrees of hip flexion and trunk rotation, respectively, and completed 18 repetitions on the right side. On the left side, participants demonstrated an average of 54.8 ± 16.8 degrees of hip flexion and 22.0 ± 8.7 degrees of trunk rotation, with a max ROM of 62.7 ± 16.3 and 26.1 ± 10.1 degrees of hip flexion and trunk rotation, respectively, and completed 17 repetitions.

**Figure 4:**
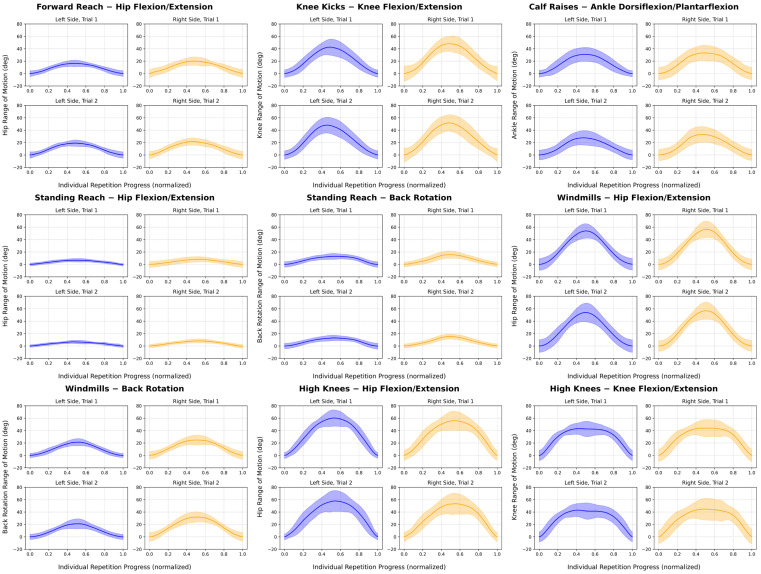
Average joint angle range of motion traces for the joints of interest from each repetition of each exercise. The solid line and shaded region represent the mean and standard deviation of the range of motion of all repetitions across all participants at a point along the normalized repetition progression. Purple indicates the left side and yellow indicates the right side.

When performing the high knees exercise on the right side, participants illustrated an average of 59.6 ± 13.5 and 46.8 ± 18.5 degrees of hip flexion and knee flexion, respectively, a max ROM of 67.9 ± 12.8 degrees of hip flexion and 56.9 ± 17.9 degrees of knee flexion and completed 21 repetitions. On the left, participants illustrated an average of 63.3 ± 8.3 and 45.6 ± 12.1 degrees of hip and knee flexion, respectively, a max ROM of 71.6 ± 9.3 degrees of hip flexion and 56.0 ± 13.2 degrees of knee flexion and completed 21 repetitions. The average joint angle traces for each exercise are given in Figure 4.

### 3.3. Physiological Variables

**Figure 5:**
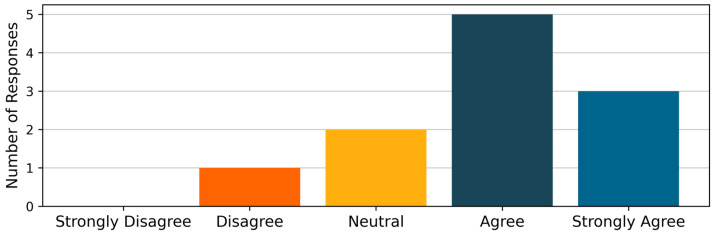
Participant responses to the statement, “I would find a system like SWS useful in my daily exercise”.

Physiologically, participants began each exercise session with an average heart rate (HR) of 85.4 ± 8.6 bpm and an RPE of 1 on a 10-point scale. After completing all six seated and standing exercises, participants recorded an average HR of 107.0 ± 11.9 and an RPE of 6.5. In addition, the average standing forward reach measured prior to exercising with SWS was 14.0 ± 3.2 inches, while post-session, it was 15.7 ± 2.1 inches (*p* < 0.01) (Table 5). Of the 11 participants, eight either agreed or strongly agreed that the system would be useful in their daily exercise, while three were either neutral or disagreed (Figure 5).

## 4. Discussion

The aim of this study was to determine if the robot-assisted exercise system, SWS, could be utilized to lead individuals with PD through ranges of motion at multiple joints, and provide an effective workout. The goal was to assess if the system had the potential to be leveraged as an in-home PT aid to encourage more consistent execution of PT exercises outside of the clinic or therapy office. Results from this study confirmed our first two hypotheses. Everyone completed the exercises as instructed and sustained a moderate workout, as measured by HR and RPE or how challenging they felt the exercises to be. Most participants also illustrated an immediate improvement in their standing forward reach from pre- to post-exercise session.

### 4.1. Joint Motion

The ROM results presented here varied by participant and by joint, as expected. For example, there was greater ROM at the hip during high knees than at the ankle during calf raises. Yet, there were not great differences in performance between the dominant and non-dominant limbs or between the more and less affected sides. In addition, a goal ROM for a given participant is going to be individual-specific. However, the robotic system was able to modulate the difficulty based on the individual and the exercise to reach a challenging, yet achievable, position. This is supported by the consistent values that were seen between trial one and trial two for each participant.

### 4.2. Balance and Mobility

This pilot study included just a single exercise session; therefore, it is currently unknown if exercising with the robotic system consistently could lead to specific improvements in joint ROM. It is also unknown if there are differences in improvements based on a participant’s dominant or more affected side over time. However, to determine if there were acute benefits of exercising with the system, which might indicate potential long-term improvements, participants completed a standing forward reach test before and immediately following the exercises. The results illustrated a statistically significant average increase of almost two inches after a single session with the SWS system. This finding is promising and indicates that exercising with the robotic system could lead to further increases in flexibility and joint mobility over time.

### 4.3. Physiological Effort

To further determine whether exercising with the robotic system was appropriately challenging, HR and RPE were also collected. By assessing both HR and RPE, we were able to measure how challenging the exercise session was, objectively (HR) and subjectively (RPE). Both measures are important to consider in the case of certain medications that may make the objective data less reliable. As anticipated, both HR and RPE were initially low prior to the beginning of participants’ sessions and climbed steadily over the course of the exercise session, as per Table 5. The average HR increased by approximately 20 beats per minute over the course of the session, while RPE increased from an average of 1 to an average of 6.5. This increase indicates that the exercises provided a moderate-to-vigorous workout for the participants. These results indicate that exercising with the SWS system could lead to improvements in both ROM and cardiovascular health.

### 4.4. Limitations

This study had limitations. Firstly, this study involved a single session; therefore, it is unknown if exercising with the system could lead to improvements in balance and ROM like those seen with longer therapy and rehabilitative programs. Future studies with a greater number of exercises over a series of weeks should be conducted to determine the long-term benefits of the robotic system for people with PD. Second, this study only utilized six exercises, while the number of exercises that could be prescribed is significantly greater. Additional exercise types are also important to alleviate boredom. Third, the iteration of SWS utilized for this study could not provide feedback to participants regarding their form or execution, which could be a limitation for in-home use. Further, individuals with PD do have a risk of falling that would need to be addressed if the system is going to be used independently. Fourth, the robotic system operates within certain parameters, so there were cases where the exercise could not be made challenging enough. For example, a taller individual may need the robot to operate at a higher height than the robot was able to reach to achieve their maximum ROM for the high knees exercise. Exercises may need to be modified accordingly in these scenarios. Lastly, the robotic system was controlled by a research team member instead of the participants themselves. This lack of a user interface is a barrier for in-home use. Future work needs to create a graphic user interface that is more user-friendly so that the system will be easy and convenient to operate.

## 5. Conclusions

To our knowledge, this is the first study to quantify ROM over the lower limbs and the trunk for people with PD when exercising with a robotic system. Results indicate that the system can lead individuals through different ROM at multiple joints while also providing a moderate workout. Additionally, participants reported enjoying the experience and most felt that the SWS robot-assisted exercise system would be valuable in their daily exercise routines. This system could be a useful addition to the home exercise routine of individuals with PD to help encourage regular activity and completion of their prescribed PT exercises. Additional work should be done to design a more user-friendly system that can be utilized by participants independently. Future studies should also work to include additional exercises and feedback options to optimize the benefits that may be gained by utilizing the system regularly. However, robot-assisted exercise systems could be an excellent solution for individuals that struggle to adhere to traditional rehabilitation programs.

## Figures and Tables

**Table 1:** Participant demographics.

Age (y)	68.3 ± 6.2
Gender	7 F, 4 M
Height (in)	67.0 ± 3.7 in
Weight (lbs)	162.8 ± 31.7
Years since Parkinson’s diagnosis	7 ± 5 years
Hand dominance	9 R, 2 L
Leg dominance	10 R, 1 L
Most affected side	5 R, 6 L
Dominant hand	9 R, 2 L
Dominant leg	10 R, 1 L

**Table 2:** Pre-assessment questionnaires and motor–cognitive test scores.

Assessment	Score
Short Form 12-Quality of Life—Mental Component Score	46.5 ± 4.5
Short Form 12-Quality of Life—Physical Component Score	49.2 ± 8.8
Activities Specific Balance Confidence Scale (/100)	85.5 ± 10.5
Physical Activity Scale for the Elderly (Total Score)	189.2 ± 108.6
PDQ-39 Questionnaire Summary Index * (/100)	17.3 ± 13.3
30 s Chair Stand (reps)	14.0 ± 3.0
Body Position Spatial Task	20.8 ± 6.9
Four-Square Step Test (s)	8.83 ± 1.49
Timed Up and Go—Simple (s)	8.44 ± 1.79
Timed Up and Go—Cognitive (s)	10.75 ± 2.71
6 min Walk Test (m)	465.7 ± 109.4

* Higher scores reflect worse quality of life; these participants with PD had some fear of falling, a generally high quality of life, were at moderate functioning and were at a low risk of falling.

**Table 3:** Joint range of motion and movement repetitions on the right.

Task	Joint Motion	Avg ROM Trial 1	Max ROM Trial 1	Reps Trial 1	Avg ROM Trial 2	Max ROM Trial 2	Reps Trial 2
Forward Reach	Trunk Flexion	21.5 ± 11.0	28.2 ± 13.0	20 ± 2	22.6 ± 8.5	32.0 ± 16.3	20 ± 3
Knee Kicks	Knee Extension	52.1 ± 22.1	63.4 ± 22.0	24 ± 3	57.3 ± 20.3	69.2 ± 22.2	23 ± 4
Calf Raises	Ankle Plantarflexion	34.0 ± 15.0	42.5 ± 13.2	21 ± 9	35.8 ± 15.7	34.4 ± 34.5	23 ± 10
Standing Reach	Hip Flexion	9.5 ± 4.3	13.7 ± 8.0	8 ± 5	9.4 ± 3.2	12.8 ± 6.1	9 ± 6
	Trunk Rotation	16.1 ± 6.4	19.1 ± 7.3	8 ± 5	15.7 ± 5.4	17.7 ± 6.0	9 ± 6
High Knees	Hip Flexion	60.6 ± 13.7	70.4 ± 9.4	22 ± 5	58.6 ± 13.9	65.5 ± 15.5	20 ± 9
	Knee Flexion	47.4 ± 18.3	60.1 ± 16.3	22 ± 5	46.2 ± 19.6	53.8 ± 19.7	20 ± 9
Windmills	Hip Flexion	59.1 ± 18.3	66.4 ± 18.0	18 ± 4	58.6 ± 18.8	65.3 ± 18.8	19 ± 5
	Trunk Rotation	25.1 ± 11.2	29.9 ± 11.9	18 ± 4	31.7 ± 11.6	37.9 ± 13.0	19 ± 5

**Table 4:** Joint range of motion and movement repetitions on the left.

Task	Joint Motion	Avg ROM Trial 1	Max ROM Trial 1	Reps Trial 1	Avg ROM Trial 2	Max ROM Trial 2	Reps Trial 2
Forward Reach	Trunk Flexion	18.3 ± 8.2	22.4 ± 8.5	20 ± 3	20.5 ± 9.5	25.2 ± 12.5	21 ± 4
Knee Kicks	Knee Extension	47.2 ± 10.9	55.7 ± 10.8	23 ± 3	53.3 ± 11.9	50.3 ± 41.3	22 ± 4
Calf Raises	Ankle Plantarflexion	36.7 ± 8.1	45.3 ± 10.2	23 ± 9	29.6 ± 15.3	37.7 ± 14.8	23 ± 11
Standing Reach	Hip Flexion	9.3 ± 3.6	15.4 ± 12.4	7 ± 5	8.2 ± 2.2	10.8 ± 4.3	7 ± 5
	Trunk Rotation	14.1 ± 5.3	16.8 ± 6.6	7 ± 5	14.4 ± 6.0	16.2 ± 6.9	7 ± 5
High Knees	Hip Flexion	63.9 ± 8.6	71.7 ± 9.8	21 ± 6	62.8 ± 8.4	71.4 ± 9.2	21 ± 7
	Knee Flexion	45.6 ± 12.8	55.0 ± 14.8	21 ± 6	45.7 ± 11.9	57.0 ± 12.1	21 ± 7
Windmills	Hip Flexion	53.8 ± 18.3	61.6 ± 17.7	18 ± 6	55.7 ± 16.1	63.8 ± 15.6	17 ± 4
	Trunk Rotation	21.5 ± 8.2	25.1 ± 9.9	18 ± 6	22.4 ± 9.6	27.0 ± 10.8	17 ± 4

**Table 5:** Physiological variables.

Assessment	Time Point 1—Pre	Time Point 2—Mid	Time Point 3—End
Heart Rate (bpm)	85.4 ± 8.6	87.2 ± 12.7	107.0 ± 11.9
RPE (10-pt)	1.3 ± 0.6	3.0 ± 1.0	6.5 ± 1.8
Standing Forward Reach (in)	14.0 ± 3.2	NA	15.7 ± 2.1

Participants increased their heart rate (HR) and rate of perceived exertion (RPE) over the session, indicating that the exercise was of moderate intensity. Participants improved their standing forward reach distance after a single session.

## Data Availability

The original contributions presented in this study are included in the article. Further inquiries can be directed to the corresponding author.
